# Incarcerated native orthotopic kidney through an inguinal hernia: a case report and literature review

**DOI:** 10.1093/jscr/rjad378

**Published:** 2023-07-12

**Authors:** Jisoo Kim, Brittany Harper, Alisan Turner, Irving Alvarado Gonzalez, Alonso Andrade

**Affiliations:** Department of Surgery, Texas Tech University Health Sciences Center, 4800 Alberta Ave, El Paso, TX 79905, USA; Department of Surgery, Texas Tech University Health Sciences Center, 4800 Alberta Ave, El Paso, TX 79905, USA; Department of Surgery, Texas Tech University Health Sciences Center, 4800 Alberta Ave, El Paso, TX 79905, USA; Department of Surgery, Texas Tech University Health Sciences Center, 4800 Alberta Ave, El Paso, TX 79905, USA

## Abstract

This is a case report of a 66-year-old male with an incarcerated inguinal hernia that contained native orthotopic kidney causing obstructive uropathy. The patient presented to the hospital due to intractable right groin pain, and CT imaging showed herniation of the right kidney in the right inguinal hernia with the upper pole squeezed in the inguinal canal. The patient underwent surgery to reduce the hernia contents and hernia repair with mesh. He was discharged on postoperative day 3 without complication. To the best of our knowledge, this is the first reported case of an incarcerated native orthotopic kidney without anatomic abnormality that had to undergo surgical intervention.

## INTRODUCTION

Inguinal hernias are a common surgical issue, and various contents have been reported including bladder, appendix, bowels, etc. [1]. These hernias can become incarcerated and represent approximately 5–15% of hernias that require surgical intervention [2]. The presence of genitourinary organs within inguinal hernias, however, is rare and most commonly involves the bladder [3]. Inguinal hernias that involve the kidney are especially unique, and literature is limited to case reports and are often in the setting of ectopic kidneys. Upon review of the literature, there are only four documented reports of an inguinal hernia containing an orthotopic kidney [4–7]. One of these cases involved a duplicate kidney and underwent operative repair of the hernia. The remaining three were treated nonoperatively [7]. Here, we present a rare case of a patient with an incarcerated inguinal hernia containing a native kidney. The patient underwent operation for reduction of hernia contents followed by hernia repair with mesh. This is the first to be reported in the literature for an incarcerated native orthotopic kidney without notable anatomic abnormalities.

## CASE REPORT

**Figure 1:**
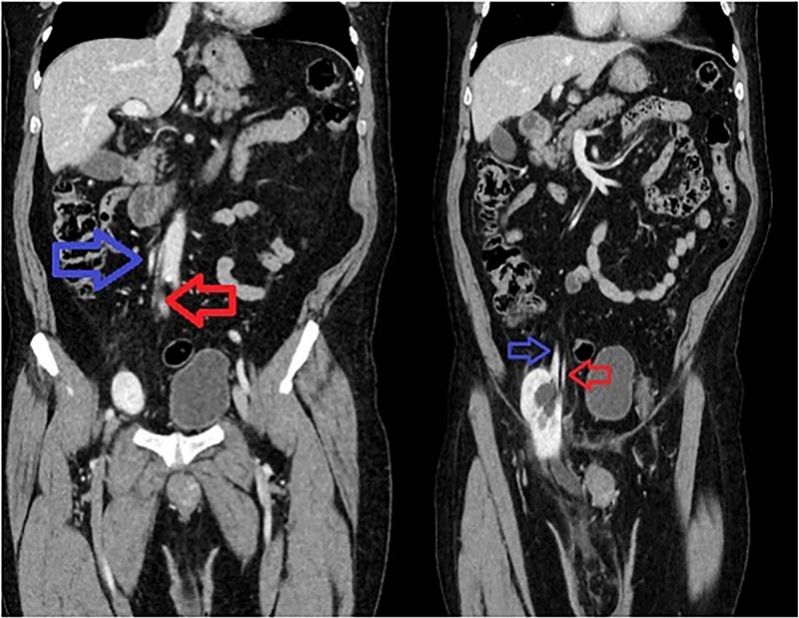
Sequential coronal CT with IV contrast. Left image showing renal artery (red) and vein (blue) arising from abdominal aorta and IVC. Right image showing descending vessels going into the incarcerated right kidney.

**Figure 2:**
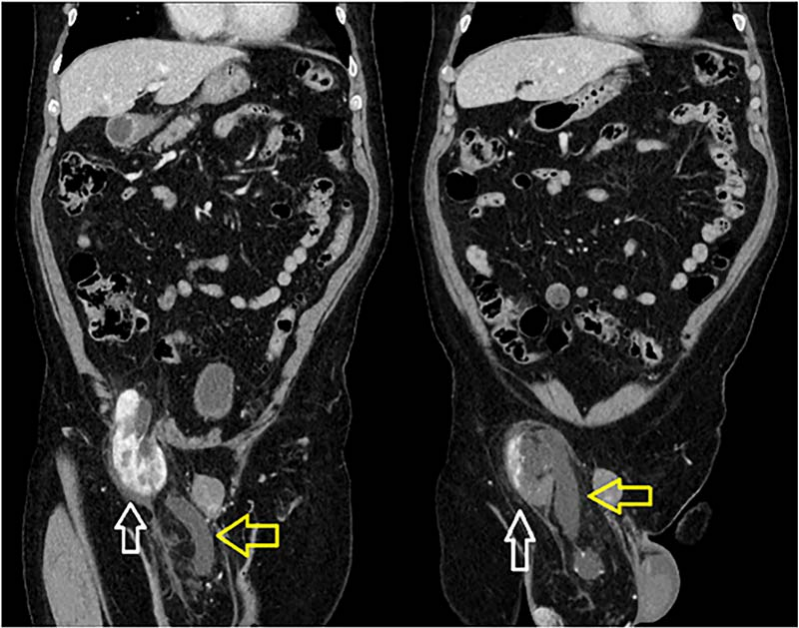
Incarcerated right kidney (white) in the inguinal hernia with distended ureter within the hernia sac (yellow).

A 66-year-old Hispanic male presented to our emergency department with 1 week of right groin pain and swelling. He came to the hospital due to worsening pain. He denied any nausea, emesis or fever. He was passing gas and having bowel movements without difficulty. He denied any issues with voiding, hematuria or urinary urgency. His past medical history includes congenital deformity of his left upper extremity and past surgical history of open left inguinal hernia repair. His BMI is 32.5. His serum creatinine was 1.40 mg/dL. He had a normal white blood cell count at 9.8 × 10^3^/UL and hemoglobin at 14.1G/DL. On examination, he had right inguinal tenderness to palpation with an obvious hernia extending to the scrotum. No skin changes were present. We were unable to reduce the hernia at bedside due to pain. A computed tomography (CT) of abdomen and pelvis with IV contrast showed herniation of the right kidney in the right inguinal hernia with the upper pole in the inguinal canal. The right renal artery and vein arose from the aorta and inferior vena cava, respectively, around the level of L1-L2 region (Fig. 1). The right ureter was incarcerated within the hernia and dilated up to 2 cm in diameter. The ureter transitions to normal caliber as it exits the hernia, concerning for obstructive uropathy (Fig. 2).

We started the operation laparoscopically. The extent of the hernia could not be well appreciated from the intraperitoneal view; since the hernia content was from the retroperitoneum. After creating a preperitoneal flap, there was a large amount of perinephric adipose tissue associated with chronic adhesions. This made it difficult to dissect the kidney out of the indirect inguinal hernia. Although the kidney was able to be skeletonized laterally, it was not safe to dissect medially due to inability to identify the renal artery, vein and pelvis. Therefore, lower midline laparotomy and right groin incisions were made. We pulled the kidney from the lower midline incision while concurrently pushing the kidney from the groin incision to safely reduce it. After reduction, we ensured that the right kidney stayed in the retroperitoneal space without tension. The kidney appeared grossly viable and the patient’s urine remained clear throughout the entirety of the case. The right groin incision allowed us to visualize the hernia defect to ensure all hernia contents including kidney, ureter and perinephric adipose tissues were reduced adequately. A Lichtenstein hernia repair with polypropylene mesh was performed because of its favorable low recurrence rate [8]. The patient had no intraoperative complications. On postoperative day (POD) 1, the Foley catheter was discontinued and the patient was tolerating his diet and ambulating. The creatinine ranged from 1.4 to 1.5 mg/dL throughout the hospitalization. The patient was discharged home on POD 3 without any issues.

## DISCUSSION

The renal artery normally arises from the aorta at the L1-L2 level, just below the origin of the SMA. In a pelvic kidney, the arterial origin varies but most often arises from the level of the aortic bifurcation or lower [9]. For our patient, the renal artery and vein arose from the aorta and inferior vena cava, respectively, at the expected level for an orthotopic kidney. Therefore, it is likely that for the current case in question, the patient’s right kidney started in the normal anatomical position and descended to the pelvis and into the inguinal hernia over time. Due to the patient’s presentation of persistent pain and obstructive uropathy with acute kidney injury, a decision was made to perform a surgical intervention urgently. A previous case of inguinal hernia containing a native kidney did not require intervention due to the patient being asymptomatic and frail [4]. Another case had a patient who underwent cystoscopy with no evidence of hydronephrosis. This case also did not perform surgery for hernia due to the patient being asymptomatic with multiple comorbidities [5]. One case of inguinal hernia containing a duplicated kidney underwent nephropexy, orchiectomy and inguinal hernia repair with mesh placement [6]. There are other reported cases of inguinal hernia containing a kidney, but these are from transplanted kidney or an ectopic pelvic kidney [10–13]. To the best of our knowledge, this is the first reported case of an incarcerated inguinal hernia containing a native orthotopic kidney without significant anatomic abnormality that required a surgical intervention.

## CONCLUSION

This is the first case of a native orthotopic kidney without significant anatomic abnormality incarcerated in an inguinal hernia that underwent surgery for reduction of hernia content and repair of hernia. The best surgical approach remains per surgeon’s discretion depending on case presentation. This patient required open surgery to reduce the kidney back into the retroperitoneum then inguinal hernia repaired with mesh. The patient was discharged on POD3 and was followed up as an outpatient 2 weeks later without any issues.

## CONFLICT OF INTEREST STATEMENT

None declared.

## FUNDING

None.
